# Genome Sequences of Two Putative Streptogramin Producers, *Streptomyces* sp. Strains TÜ 2975 and TÜ 3180, from the Tübingen Strain Collection

**DOI:** 10.1128/MRA.01582-19

**Published:** 2020-05-21

**Authors:** Oliver Hennrich, Franziska Handel, Regina Ort-Winklbauer, Yvonne Mast

**Affiliations:** a Department of Microbiology/Biotechnology, Interfaculty Institute of Microbiology and Infection Medicine, Faculty of Science, Eberhard Karls University of Tübingen, Tübingen, Germany; b German Center for Infection Research (DZIF), Partner Site Tübingen, Tübingen, Germany; c Department of Bioresources for Bioeconomy and Health Research, Leibniz Institute DSMZ–German Culture Collection for Microorganisms and Cell Cultures, Braunschweig, Germany; d Institute for Microbiology, Technical University of Braunschweig, Braunschweig, Germany; University of Maryland School of Medicine

## Abstract

*Streptomyces* sp. TÜ 2975 and TÜ 3180 are two strains from the Tübingen *Actinomycetes* strain collection. Here, we present the draft genome sequences of TÜ 2975 and TÜ 3180, with sizes of 7.62 Mb and 8.63 Mb, respectively.

## ANNOUNCEMENT

Streptogramin antibiotics such as pristinamycin and griseoviridin/viridogrisein are valuable drugs used in human medicine and agriculture which act as protein synthesis inhibitors by binding to the 50S subunit of the bacterial ribosome (1). In the context of screening strains from the Tübingen *Actinomycetes* strain collection (https://uni-tuebingen.de/fakultaeten/mathematisch-naturwissenschaftliche-fakultaet/fachbereiche/interfakultaere-einrichtungen/imit/technologien/natresource/) for antibiotics that target bacterial protein synthesis, *Streptomyces* sp. strain TÜ 2975 and *Streptomyces* sp. strain TÜ 3180 were identified. Here, we present the annotated genome sequences of both strains and report on their genetic potential to produce streptogramin antibiotics.

For DNA isolation, TÜ 2975 and TÜ 3180 cells were cultivated for 2 days in 50 ml of R5 medium (2) at 30°C. For cell lysis, lysozyme (10 mg/ml; Serva) and achromopeptidase (5 mg/ml; Sigma) were added as reported previously (3). Genomic DNA was extracted and purified using the Genomic-tip 100/G kit from Qiagen (catalog number 10243). The genomic DNA isolation procedure was carried out following the standard protocol provided by the manufacturer. For genome sequencing, a single SMRTbell library was prepared according to the Pacific Biosciences sample preparation protocol (https://www.pacb.com/wp-content/uploads/2015/09/User-Bulletin-Guidelines-for-Preparing-20-kb-SMRTbell-Templates.pdf), and sequencing was performed with the PacBio RS II platform. The genomes were assembled with Hierarchical Genome Assembly Process (HGAP) v3.0 (4). HGAP data processing consisted of PreAssembler v1 for filtering, PreAssembler v2 and AssembleUnitig v1 for assembly (4), BLASR v1 (5) for mapping, and Quiver v1 (4) for consensus polishing using only unambiguously mapped reads. HGAP3 settings were kept at their defaults, except for the genome size estimate parameter, which was set to 8.0 Mbp. For TÜ 2975, 136,147 filtered reads with an *N*_50_ value of 10,822 bp were assembled into one contig, yielding a 7,623,788-bp draft sequence with a coverage depth of 87× and an average G+C content of 71.04%. The average read length was 7,111 bp. Genome annotation was performed with the NCBI Prokaryotic Genome Annotation Pipeline (PGAP) software tool v4.6 (6), yielding 6,950 coding sequences (CDSs), 80 tRNAs, and 18 rRNAs. For TÜ 3180, 146,177 filtered reads with an *N*_50_ value of 13,352 bp were assembled into two contigs, yielding an 8,634,962-bp draft sequence with a coverage depth of 95× and an average G+C content of 72.97%. The average read length was 9,812 bp. Genome annotation was performed with PGAP v4.6 (6), yielding 7,470 CDSs, 97 tRNAs, and 18 rRNAs.

Using 16S marker genes, EzTaxon v2.1 (7) identified TÜ 2975 as most similar to Streptomyces xantholiticus NBRC13354^T^, with 99.86% similarity (8, 9). TÜ 3180 showed 99.38% similarity to Streptomyces carpiensis NBRC14214^T^ (10). The Type (Strain) Genome Server (TYGS) v1.0 (11) was applied to conduct phylogenomic analyses based on full-length genome sequences. It was found that TÜ 2975 is related to the type strain Streptomyces lunaelactis DSM 42149 (12), and TÜ 3180 is similar to the type strain Streptomyces ghanaensis ATCC 14672 (13, 14), with digital DNA-DNA hybridization (dDDH) values (formula *d_4_*) of 26% and 44%, respectively. For all software analyses, default settings were used.

In order to identify biosynthetic gene clusters (BGCs), the TÜ 2975 and TÜ 3180 genome sequences were analyzed with antiSMASH v4.0 (15). For TÜ 2975, antiSMASH predicted 20 BGCs, and 1 cluster shows >60% similarity to a known gene cluster encoding pristinamycin (16). For TÜ 3180, antiSMASH predicted 27 BGCs, and 1 cluster shows >70% similarity to a known cluster encoding griseoviridin/viridogrisein (17). Thus, TÜ 2975 and TÜ 3180 host the genetic potential to synthesize the streptogramin antibiotics pristinamycin and griseoviridin/viridogrisein, respectively.

### Data availability.

This whole-genome shotgun project has been deposited at GenBank under the accession numbers CP047140 (TÜ 2975) and WOXS00000000 (TÜ 3180). The raw sequencing data are available under SRA accession numbers SRX7351729 (TÜ 2975) and SRX7351340 (TÜ 3180).

## References

[1] Mast Y, Wohlleben W 2014 Streptogramins—two are better than one! Int J Med Microbiol 304:44–50. doi:10.1016/j.ijmm.2013.08.008. 24119565

[2] Kieser T, Bibb MJ, Buttner MJ, Chater KF, Hopwood DA 1985 Practical *Streptomyces* genetics. John Innes Foundation, Norwich, England.

[3] Jiao J-Y, Carro L, Liu L, Gao X-Y, Zhang X-T, Hozzein WN, Lapidus A, Huntemann M, Reddy TBK, Varghese N, Hadjithomas M, Ivanova NN, Göker M, Pillay M, Eisen JA, Woyke T, Klenk H-P, Kyrpides NC, Li W-J 2017 Complete genome sequence of *Jiangella gansuensis* strain YIM 002^T^ (DSM 44835^T^), the type species of the genus *Jiangella* and source of new antibiotic compounds. Stand Genomic Sci 12:21. doi:10.1186/s40793-017-0226-6. 28174619 PMC5292007

[4] Chin C-S, Alexander DH, Marks P, Klammer AA, Drake J, Heiner C, Clum A, Copeland A, Huddleston J, Eichler EE, Turner SW, Korlach J 2013 Nonhybrid, finished microbial genome assemblies from long-read SMRT sequencing data. Nat Methods 10:563–569. doi:10.1038/nmeth.2474. 23644548

[5] Chaisson MJ, Tesler G 2012 Mapping single molecule sequencing reads using basic local alignment with successive refinement (BLASR): application and theory. BMC Bioinformatics 13:238. doi:10.1186/1471-2105-13-238. 22988817 PMC3572422

[6] Tatusova T, DiCuccio M, Badretdin A, Chetvernin V, Nawrocki EP, Zaslavsky L, Lomsadze A, Pruitt KD, Borodovsky M, Ostell J 2016 NCBI Prokaryotic Genome Annotation Pipeline. Nucleic Acids Res 44:6614–6624. doi:10.1093/nar/gkw569. 27342282 PMC5001611

[7] Yoon S-H, Ha S-M, Kwon S, Lim J, Kim Y, Seo H, Chun J 2017 Introducing EzBioCloud: a taxonomically united database of 16S rRNA gene sequences and whole-genome assemblies. Int J Syst Evol Microbiol 67:1613–1617. doi:10.1099/ijsem.0.001755. 28005526 PMC5563544

[8] Konev IE, Tsyganov VA 1962 A new species in the yellow actinomycetes group, Actinomyces xantholiticus n. sp. Mikrobiologiia 31:1023–1028. (In Russian.) 14034575

[9] Pridham TG 1970 New names and new combinations in the order Actinomycetales Buchanan 1917. U.S. Department of Agriculture, Washington, DC.

[10] Goodfellow M, Williams ST, Alderson G 1986 Transfer of *Chainia* species to the genus *Streptomyces* with emended description of species. Syst Appl Microbiol 8:55–60. doi:10.1016/S0723-2020(86)80148-5.

[11] Leibniz Institute DSMZ. Type (strain) genome server. https://tygs.dsmz.de/.

[12] Maciejewska M, Pessi IS, Arguelles-Arias A, Noirfalise P, Luis G, Ongena M, Barton H, Carnol M, Rigali S 2015 *Streptomyces lunaelactis* sp. nov., a novel ferroverdin A-producing *Streptomyces* species isolated from a moonmilk speleothem. Antonie Van Leeuwenhoek 107:519–531. doi:10.1007/s10482-014-0348-4. 25491121

[13] Linder F, Wallhausser KH, Huber G July 1972 Moenomycin and process for producing same. U.S. patent 3,674,866A.

[14] Skerman VBD, McGowan V, Sneath PHA 1980 Approved lists of bacterial names. Int J Syst Evol Microbiol 30:225–420. doi:10.1099/00207713-30-1-225.

[15] Blin K, Wolf T, Chevrette MG, Lu X, Schwalen CJ, Kautsar SA, Suarez Duran HG, de Los Santos ELC, Kim HU, Nave M, Dickschat JS, Mitchell DA, Shelest E, Breitling R, Takano E, Lee SY, Weber T, Medema MH 2017 antiSMASH 4.0—improvements in chemistry prediction and gene cluster boundary identification. Nucleic Acids Res 45:W36–W41. doi:10.1093/nar/gkx319. 28460038 PMC5570095

[16] Mast Y, Weber T, Gölz M, Ort-Winklbauer R, Gondran A, Wohlleben W, Schinko E 2011 Characterization of the “pristinamycin supercluster” of *Streptomyces pristinaespiralis*. Microb Biotechnol 4:192–206. doi:10.1111/j.1751-7915.2010.00213.x. 21342465 PMC3818860

[17] Xie Y, Wang B, Liu J, Zhou J, Ma J, Huang H, Ju J 2012 Identification of the biosynthetic gene cluster and regulatory cascade for the synergistic antibacterial antibiotics griseoviridin and viridogrisein in *Streptomyces griseoviridis*. Chembiochem 13:2745–2757. doi:10.1002/cbic.201200584. 23161816

